# Beyond conventional pollinators: advancing diversity assessments of neglected insect taxa

**DOI:** 10.1093/aesa/saag025

**Published:** 2026-08-05

**Authors:** Aleida Ascenzi, Lorenzo Marini, Costanza Geppert, Vivian Feng, Enrico Rosso, Rudolf Meier, Pierfilippo Cerretti

**Affiliations:** Department of Biology and Biotechnologies “Charles Darwin”, Sapienza University of Rome, Rome, Italy; Museum of Zoology (MZUR), Sapienza University of Rome, Rome, Italy; Department of Agronomy, Food, Natural Resources, Animals and Environment, (DAFNAE), University of Padua, Legnaro, Italy; Department of Agronomy, Food, Natural Resources, Animals and Environment, (DAFNAE), University of Padua, Legnaro, Italy; Museum für Naturkunde, Center for Integrative Biodiversity Discovery, Leibniz-Institut für Evolutions-und Biodiversitätsforschung, Berlin, Germany; Institut für Biologie, Humboldt University Berlin, Berlin, Germany; Eurac Research, Bolzano, Autonome Provinz Bozen, Südtirol, Italy; Museum für Naturkunde, Center for Integrative Biodiversity Discovery, Leibniz-Institut für Evolutions-und Biodiversitätsforschung, Berlin, Germany; Institut für Biologie, Humboldt University Berlin, Berlin, Germany; Department of Biology and Biotechnologies “Charles Darwin”, Sapienza University of Rome, Rome, Italy; Museum of Zoology (MZUR), Sapienza University of Rome, Rome, Italy

**Keywords:** coastal dunes, COI, dark taxa, ecological guild, hand net, megabarcoding, pan trap

## Abstract

Ideally, biodiversity surveys would sample insect assemblages randomly and without taxonomic bias, yet most protocols still prioritize conspicuous, charismatic, or economically important taxa (eg pollinators). Consequently, Diptera, one of the most species-rich and ecologically important insect orders, remain largely overlooked, with only a few families like Syrphidae routinely included in biodiversity assessments. To address this imbalance, here we tested a protocol designed to better capture the diversity of non-charismatic dipteran groups by comparing hand-netting and pan traps and by overcoming taxonomic impediments with cost-efficient DNA “megabarcoding” using non-destructive DNA extraction and third-generation sequencing. We applied it to brachyceran fly (Diptera: Brachycera) assemblages across four Mediterranean coastal habitats, key for coastal protection and tourism yet poorly documented. We clustered 13,877 cytochrome c oxidase subunit I barcodes into 823 molecular operational taxonomic units (MOTUs) at a 2% distance threshold, more than 10% of all Diptera species recorded for Italy. MOTUs were assigned to 48 families by morphological inspection of a single representative specimen. We then assigned ecological guilds at the family level, enabling joint assessment of taxonomic and functional diversity. Pan traps collected twice as many specimens and reached higher sample coverage than transect counts, while total MOTU richness was similar. However, assemblages differed in composition, indicating that the two methods are complementary, and still shared 27% to 40% of ecological-guild representation across habitats. Our integrative discovery framework reveals outstanding Diptera diversity and provides a scalable framework combining multiple sampling techniques with high-throughput molecular workflows, applicable to other neglected insect taxa and ecosystems.

## Introduction

Insects are an extraordinarily species-rich and functionally diverse clade, with lower-bound global estimates being 5.5 million species (Stork 2018). Yet, knowledge remains highly fragmented, skewed toward charismatic or economically important groups, while hyperdiverse but inconspicuous lineages are still severely neglected (Habel et al. 2021, Wang et al. 2021). These gaps are further amplified by methodological and geographic biases (Cardoso and Leather 2019, Sánchez-Fernández et al. 2021), which distort estimates of species abundance and distribution and thereby weaken the reliability of biodiversity assessments and conservation strategies.

Among insect orders, Diptera epitomize this imbalance, representing a case where extraordinary richness, accounting for approximately 10% of the world’s described biodiversity (Marshall 2012, Smith and Mayfield 2015), and ecological importance (Orford et al. 2015, Hebert et al. 2016, Srivathsan et al. 2023), sharply contrast with their systematic underrepresentation in biodiversity surveys. Pollinator diversity has attracted substantial attention in the last few decades, with Syrphidae receiving particularly intensive study (Dousti and Hayat 2006, Montoya et al. 2012, Marín-Armijos et al. 2017, Barkalov and Mutin 2019, Speight 2024). However, institutions have recently emphasized the inclusion of “potential pollinators” in monitoring programs. For instance, the European Commission (2023) explicitly calls for investigating pollination ecology “beyond […] hoverflies,” and the EU Pollinator Monitoring Scheme (PoMS) (Potts et al. 2024) recommends prioritizing neglected dipteran groups. Diptera are among the largest “dark taxa,” lineages that comprise many thousands of species, whose species level diagnostics are poorly characterized and hard to resolve through traditional morphological methods (Page 2016, Hartop et al. 2022, Awad et al. 2025). The reason may be taxonomic impediments that are particularly pronounced (Chimeno et al. 2022, Srivathsan et al. 2023, Hartop et al. 2024, Riccardi and Hartop 2024) due to high species richness, the presence of numerous cryptic lineages (Biswas et al. 2025, Katoh et al. 2025), and the intrinsic limitations of morphology-based methods when dealing with large numbers of specimens.

In recent years, high-throughput molecular approaches have offered powerful alternatives to traditional morphospecies sorting, thus improving our ability to delimit species, clarifying phylogenetic relationships, and enhancing the sampling of overlooked taxa (Chimeno et al. 2023, Germann et al. 2011, Rodrigues and Galati 2023, Hebert et al. 2025). Among these, DNA “megabarcoding” has proven particularly effective (Meier et al. 2025). Supported by non-destructive DNA extraction and nextgeneration sequencing (NGS), it applies a reverse workflow in which all specimens are first sequenced and clustered into molecular operational taxonomic units (MOTUs), while morphological validation is carried out only a posteriori on a representative subset (Srivathsan et al. 2019, Hartop et al. 2022, Caruso et al. 2024, Meier et al. 2024). This approach bypasses time-consuming morpho-species sorting (Riedel et al. 2013, Wang et al. 2018), generating MOTUs that can be treated as species proxies (Ratnasingham and Hebert 2007, Hausmann et al. 2020, Chimeno et al. 2022) for ecological analysis (D’Souza et al. 2021, Yeo et al. 2021). It also enables the rapid processing of large samples, generating robust baselines for biodiversity surveys even in groups suffering from severe taxonomic impediments.

Numerous field methods are available for surveying dipteran diversity (Leather 2008, Yi et al. 2012, Borkent et al. 2018), with Malaise traps widely used for large-scale, passive, and standardized sampling (Mason et al. 2006, Stireman et al. 2012, Geiger et al. 2016, Karlsson et al. 2020). However, besides posing logistical challenges, ie high per-unit costs (∼€100 to 500), high conspicuity (difficult to conceal from people or livestock), and unsuitability to be placed within some habitats (eg windy areas), Malaise traps exhibit collection biases related to trap configuration (eg location, positioning, orientation) or local climatic conditions (Hutcheson 1990, Irvine and Woods 2007, Uhler et al. 2022). For instance, small-bodied specimens tend to be collected in larger numbers (Srivathsan et al. 2023), whereas taxa flying very low or high in the canopy may be under-sampled. Thus, these traps preferentially capture certain dipteran groups, introducing substantial taxonomic bias when used alone, a pattern reported across all climatic regions and habitat types (Srivathsan et al. 2023). Malaise traps can fail to capture fine-scale community heterogeneity (Favret et al. 2019), indicating that alternative methods may be preferable, especially at small spatial scales.

Sampling efficiency usually varies by target group, ie different families and ecological guilds are preferentially collected by different methods (Missa et al. 2009, O’Connor et al. 2019, Hutchinson et al. 2022). Transect-based observations and hand-netting are widely used to target flower-visiting insects (Chacoff et al. 2012, Habel et al. 2019) or parasitic insects (Yi et al. 2012, Shweta and Rajmohana 2016), but when target taxa are not directly or indirectly attracted to flowers, transects are ineffective and alternative methods are needed. Yellow pan traps efficiently sample a high diversity of insects (Disney et al. 1982, Leong and Thorp 1999) and multiple ecological guilds (Kirk 1984, Leksono et al. 2005); they are inexpensive and enable standardized, high-volume sampling with minimal expertise (Westphal et al. 2008). While protocols for pollinators are well established (Breeze et al. 2021, Grange et al. 2021, Potts et al. 2024), combined methods for sampling other dipterans remain less frequently explored in large-scale biodiversity assessments. Comparing commonly employed approaches is essential for developing effective biodiversity monitoring protocols (Chao et al. 2021, Larson et al. 2021).

In this study, we investigated dipteran communities, focusing on brachyceran flies (Diptera: Brachycera), across 4 Mediterranean coastal habitats to evaluate the effectiveness of a large-scale protocol for surveying dipterans. We employed 2 commonly used sampling methods, yellow pan traps and hand-netting, while we avoided Malaise traps because they are likely to be damaged by tourism and strong winds. We then applied a megabarcoding pipeline to efficiently generate cytochrome c oxidase subunit I (COI) sequences. Approximately 14,000 specimens were processed using a non-destructive DNA extraction protocol, with family-level assignments confirmed through morphological inspection of one representative per MOTU. Specimens were subsequently assigned to ecological guilds, and taxonomic and ecological guild diversity were assessed through a comparative analysis of the 2 sampling methods. Our investigation addressed 2 main research questions: (i) To what extent can a high-throughput megabarcoding protocol provide reliable and efficient estimates of dipteran diversity across environmental gradients? (ii) How do pan traps and hand-netting differ and complement each other in capturing taxonomic and ecological guild diversity across different habitat types when several hyperdiverse dipteran families are simultaneously considered? This study highlights the potential of standardized, large-scale protocols integrated with NGS techniques to advance biodiversity assessments and ecological studies of small-bodied insect groups usually neglected in ecological and conservation studies.

## Materials and Methods

### Study Sites

**Fig. 1.**
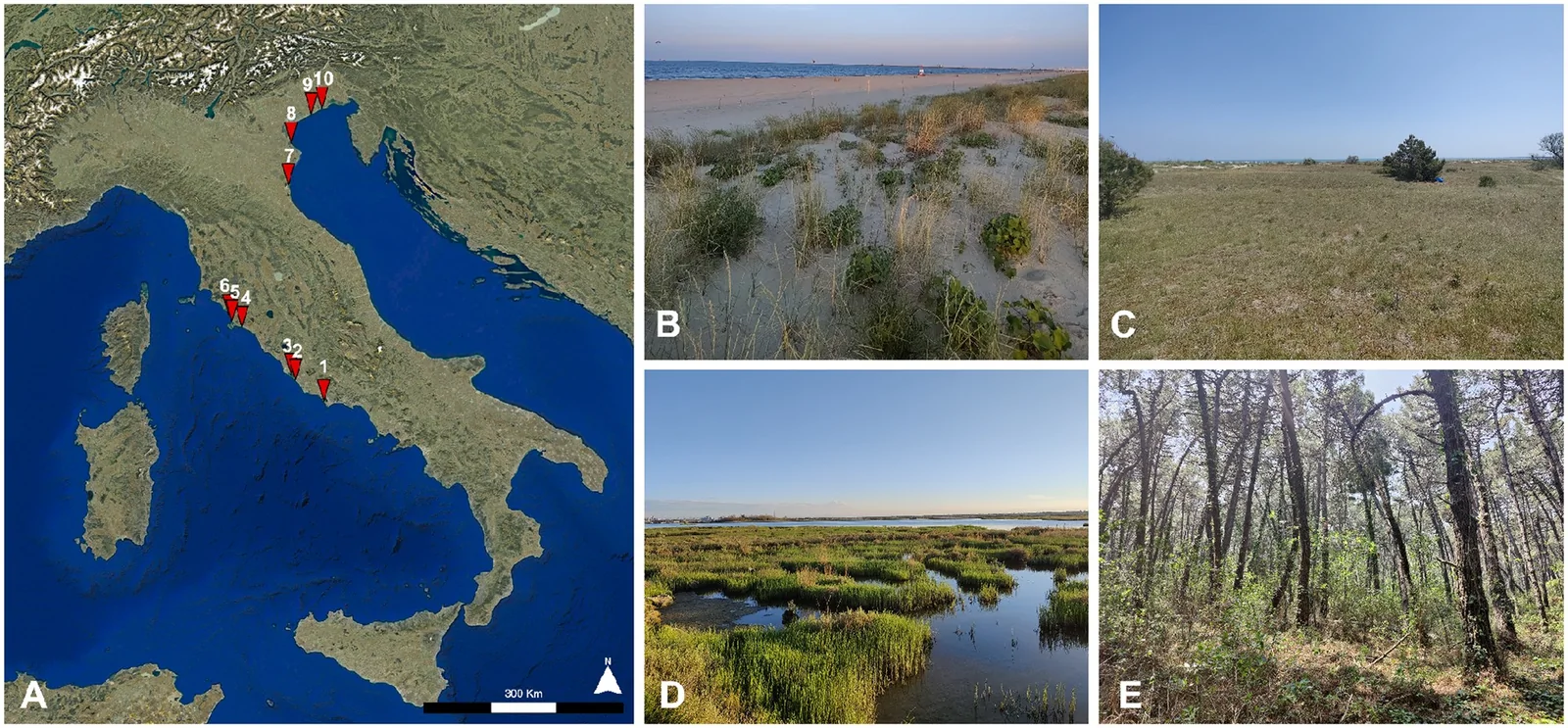
A) Map of the 10 sampling sites numbered 1 to 10 (see main text for site toponyms). Examples of surveyed coastal habitat types (site of Marina Romea, number 7 of Fig. 1A): B) mobile dunes, C) fixed dunes, D) retrodunal wetland, E) retrodunal forest (pinewood).

Sampling was conducted in 2022 across 10 sites along the Italian peninsula, representing coastal sand dune habitats located on both the Tyrrhenian and Adriatic coasts (Fig. 1). All sites were part of the Natura 2000 network.

The Tyrrhenian sites include Sabaudia, Capocotta, Castelporziano, Lago di Burano, Orbetello, and Marina di Grosseto (in order, numbers 1 to 6 of Fig. 1A). These sites are representative of low-energy shorelines typical of the Mediterranean basin and fall within the Hot-summer Mediterranean climate zone (*Csa*). In 2022, nearby meteorological stations recorded a mean annual temperature of 15.52 °C (SD: ±0.47 °C) and an annual rainfall of 750.70 mm (SD: ±78.57 mm) (Supplementary Table S1). The Adriatic sites comprise Marina Romea, Rosolina Mare, Valle Ossi, and Valle Vecchia (in order, numbers 7 to 10 of Fig. 1A). They are situated along the northeastern coast, within the Venice Lagoon system, a shallow microtidal basin-oriented NE–SW. The coasts are influenced by the northernmost and coldest waters of the Mediterranean Sea and belong to the Humid Subtropical climatic zone (*Cfa*). In 2022, these sites recorded a mean annual temperature of 13.25 °C (SD: ±0.66 °C) and an average annual rainfall of 665.35 mm (SD: ±73.92 mm) (Supplementary Table S1).

Across both coasts, the 10 sandy coastal sites represent replicated sequences of 4 habitats, identified according to the EU Habitats Directive (1992):

- shifting dunes (ie embryo dunes, foredunes, yellow dunes) (hereafter “mobile dunes”), dominated by pioneer plant communities of the drift line such as *Echinophora spinosa* L., *Calamagrostis arenaria* L., *Salsola kali* L., *Cakile maritima* Scop., *Anthemis maritima* L.;
- fixed dunes (grey dunes), defined by perennial xerophilous grasslands (eg *Oenothera biennis* L.) and/or shrublands (eg *Phillyrea angustifolia* L. and *Phillyrea latifolia* L., *Juniperus oxycedrus* L., *Cistus creticus* L., *Pistacia lentiscus* L.);
- retrodunal wetland, with salt marsh species like *Limonium narbonense* Mill., *Halimione portulacoides* (L.) Aellen, *Salicornia procumbens* Sm., and *Salicornia perennans* Willd.;
- retrodunal woodland, dominated by pinewoods (eg *Pinus pinea* L., *Pinus pinaster* Aiton) or holm oak woods (eg *Quercus ilex* L.).

### Insect Sampling Methodology

We conducted 3 sampling rounds across the 10 sites during spring and summer (Supplementary Table S1). During each round, we visited all sites on sunny, warm days within the shortest possible time frame (ie 11 to 13 consecutive days) to avoid any bias related to seasonality (note that rainfall between late April and early May 2022 expanded the duration of round 1). This resulted in a total of 30 days of sampling (ie 10 sites surveyed during 3 rounds). In each site, we identified 4 sampling plots along non-linear transects, 1 within each of the 4 coastal habitats: (i) shifting dunes; (ii) fixed dunes; (iii) retrodunal wetland; and (iv) retrodunal woodland. Sampling plots’ area was on average 1,216.45 m^2^ (SD: ±771.07 m^2^); the shape of the plots followed landscape conformation and habitat limits.

We collected flying insects using yellow pan traps (15 cm diameter, 8 cm deep, 500 ml capacity) filled to two-thirds with water and 3% biodegradable dishwashing soap to reduce surface tension, as well as hand nets. Within each sampling plot, 15 pan traps were deployed at 8:00 AM and remained active for 8 h. Pan traps were placed 5 to 8 m apart to cover the entire plot, ensuring coverage of different microhabitats. Pan traps were consistently placed in the same locations during each sampling round. Hand sampling was conducted using a hand net (30 cm diameter, 1.80 m pole) during 30-min sessions per plot, standardized by habitat type: shifting dunes (10:00 to 10:30 AM), fixed dunes (11:00 to 11:30 AM), woodlands (12:00 to 12:30 PM), and wetlands (3:00 to 3:30 PM). The operator walked the entire area, capturing flying insects and sweeping foliage during inactivity. Samples were preserved in 80% denatured ethanol, then transferred to 96% non-denatured ethanol for final storage.

### Presorting to Putative Species with MinION Barcodes

The specimens were processed following the protocol for megabarcoding used by Srivathsan et al. (2021), which employs a non-destructive DNA extraction protocol and NGS techniques. We used 96-well polypropylene plates to process 95 specimens at once (1 well was used as a negative control). We processed a total of 166 plates, of which 4 contained previously failed specimens, which were re-extracted, and 7 were only partially filled. We processed specimens of varying sizes (approximately 3 to 30 mm in length). DNA was extracted non-destructively by submerging the whole specimen (if <5 mm long) or 1 leg (if >5 mm long) in 10, 15, or 20 μl HotSHOT lysis buffer (Truett et al. 2000), depending on specimen size. We heated the buffer to leach DNA in a thermocycler with a 2-cycle protocol: 1 of 18 min at 65 °C and 1 of 2 min at 98 °C. We then added equal amounts of neutralizing buffer. We amplified 418 bp fragments of COI gene. DNA templates were amplified using forward primer BF3 CCHGAYATRGCHTTYCCHCG (Elbrecht et al. 2019) and reverse primer BR2 TCDGGRTGNCCRAARAAYCA (Elbrecht and Leese 2017). The primers were tagged with 9 bp sequences at the 5′ end (Meier et al. 2016). This procedure allows the multiplexing of >9,200 products (96 × 96 combinations) in a single flow cell. PCR products were obtained in 7 μl of Mastermix (CWBio), 1 μl each of 10 μM F and R primers, and 4 μl of template DNA (for specimens longer than 5 mm, which provide a higher concentration of DNA, 2 μl of DNA were diluted with 2 μl of purified type I water). The cycling conditions of the PCRs performed were the following: 1 initial denaturation at 95 °C for 15 min, followed by 25 cycles of denaturation at 94 °C for 30 sec, annealing at 50 °C for 1:30 min, and extension at 72 °C for 1:30 min. This was followed by a final extension cycle at 72 °C for 10 min. Amplification success was tested via gel electrophoresis (on a 1% agarose gel) for a selection of 12 wells of each plate and the negative control. PCR products of each individually labeled amplicon were pooled in equal amounts (6 μl/well). DNA was cleaned using magnetic beads (CleanNA, Waddinxveen, Netherlands) and quantified through Qubit assays. The DNA library was constructed using the Nanopore ligation kit (SQK-LSK114) and the NEBNext MinION Companion Module (E7180). Products were sequenced on R10.4.1 flow cells using the MinION Mk1C and GridION platforms (Oxford Nanopore Technologies).

### Bioinformatic and Taxonomic Workflow

Fast5 files were basecalled with the Super-accurate model using Guppy 2.3.5 + 53a111f. Demultiplexing was conducted with ONTbarcoder_0.1.12 (Windows) (Srivathsan et al. 2021). The demultiplexed reads were aligned with MAFFT v7 (Katoh and Standley 2013). All COI sequences generated in this study were deposited in GenBank under accession numbers PX533992 to PX547865 and are available at https://doi.org/10.5281/zenodo.17590749. The COI barcodes were clustered at 3 uncorrected p-distance thresholds (2%, 3%, and 4%) following common practice in the literature (Ratnasingham and Hebert 2013). To cluster the sequences to putative MOTUs, we used the Objective Clustering algorithm (Meier et al. 2006) implemented in a Python script (https://github.com/asrivathsan/obj_cluster). Other delimitation algorithms, which usually yield comparable results, are not explored further here (Yeo et al. 2018, Meier et al. 2022).

Sequences were matched to NCBI-nt (National Center for Biotechnology Information (NCBI) 1988) and BOLD (Barcode of Life Data System) (Ratnasingham and Hebert 2007) through the locally downloaded mkCOInr database (Meglécz 2023, updated as of June 2024) to get an indication of their taxonomic family. We obtained 20 target sequences for each query barcode, with a minimum e-value of 1e–5 (alignment score). We then assigned taxonomy to each barcode using readsidentifier v1.1.2 (Srivathsan et al. 2015), selecting only the best single-end hit based on alignment identity (ParseMethod = identity). Contaminants were removed from the dataset; these included barcodes with >90% similarity to non-target taxa outside Diptera Brachycera and sequences derived from cross-contaminated specimens identified through morphological inspection.

Specimens were individually stored in glass vials, which were grouped into plastic bags at haplotype distances <5% and further subdivided into sub-bags at <1% similarity. This hierarchical lumping strategy facilitates future tests of congruence between MOTUs and morphology (Srivathsan et al. 2019). Finally, family-level identification inferred from NCBI and BOLD was validated through morphological inspection of a randomly selected specimen from each 2% cluster, using dichotomous keys from Oosterbroek (1998) and Marshall (2012). In parallel, we quantified the number of query barcodes, unique haplotypes, and MOTUs incorrectly assigned to a family by BLAST analysis. Although Rhiniinae and Rhinophorinae have recently been reclassified as subfamilies within the enlarged Calliphoridae, their morphological, functional, and ecological distinctions support our decision to analyze the three separately in this study. All specimens inspected (one randomly selected specimen per molecular cluster) were photographed to implement the approach of Shirali et al. (2026); highly damaged specimens (ca. 4%) were excluded. For this purpose, we estimated specimen linear body length using Oriented Bounding Boxes (OBB). Body length was used as a proxy of body mass (see Shirali et al. 2026 for validation tests). Images are available at https://doi.org/10.5281/zenodo.17590749. All arthropods collected during the sampling campaigns are preserved in ethanol and stored at the Museum of Zoology, Sapienza University of Rome (MZUR), Italy.

### Analysis of Diversity

All statistical analyses were performed in R v. 4.4.1 (R Core Team 2022). Graphs were generated with the ggplot2 package v. 3.5.1 (Wickham 2016). We produced community matrices through a Python script (*clusterlist_to_commatrix.py*, see Yeo et al. 2021) that clusters barcodes of a FASTA format file using a priori selected p-distance thresholds. We obtained matrices for 2% distance clusters, which we considered proxies of species for all the analyses that follow. To test the confidence of the threshold, we produced clusters at 3% and 4% p-distance and tested the correlation of MOTU richness obtained at a sampling plot level with the 3 different thresholds, function *cor()* in R.

We analyzed abundance (ie number of individuals) and species richness (ie number of MOTUs) across the 4 habitat types surveyed and 2 sampling methods at multiple levels: overall diversity, family-level diversity, and ecological guilds (ie groups of taxa exploiting similar resources at the adult stage). The 10 sampling sites were treated as replicates, and community diversity was assessed using the cumulative data from all specimens collected over the 3 sampling rounds. To compare samples collected using pan traps and hand-netting across the 4 surveyed habitats, we assessed beta diversity. We employed MOTU presence–absence data and used the *beta.pair* function from the betapart package in R (v. 1.6) (Baselga and Orme 2012), which was then partitioned into turnover and nestedness components (MOTU replacement and subset-related dissimilarity, respectively).

We studied sample coverage and sample completeness from each habitat type (for sampling rounds 1 + 2 + 3) using the package iNEXT (v. 3.0.1) (Hsieh et al. 2016), plotted using the *ggiNEXT* function. To ensure more accurate estimates of species richness with reduced sampling effort, we compared the assemblages using coverage-based rarefaction and extrapolation (Chao and Jost 2012).

To investigate patterns of similarity among fly assemblages across sampling sites and habitat types, we performed a Non-metric Multidimensional Scaling (NMDS) ordination based on MOTU abundance data. Community dissimilarities were calculated using the Bray–Curtis index, and the analysis was implemented with the *metaMDS* function in the vegan R package (Oksanen et al. 2022). We further evaluated whether geographic distance contributed to differences among assemblages by applying Multiple Regression on distance matrices (MRM). This analysis was conducted using the ecodist R package (Goslee and Urban 2007). We used Bray–Curtis dissimilarities and Euclidean distances derived from the geographic coordinates of the 40 sampling plots; the regression was tested using 999 permutations.

We assigned ecological guilds to specimens based on the family ecological role or diet, referring to the adult stage: pollinators (exclusive flower visitors), potential pollinators (non-exclusive flower visitors, families that can provide pollination but also fulfill other ecological roles), phytophages, predators, hematophages, detritivores (including coprophages and saprophages), and “others” (ie secretophages, fungivores, or those with unknown roles), as the identification per MOTU limited the ability to perform a detailed ecological classification at the species level (Supplementary Table S2). We assigned guilds according to the predominant ecological roles at the family level, excluding rare cases where individual species or genera fulfill additional ecological roles. Specimens of highly speciose families, whose subgroups play different ecological roles, were assigned to multiple guilds. The classification was informed by Papp and Darvas (1997–2000), Oosterbroek (1998), and Marshall (2012). Finally, we observed functional diversity patterns across sampling methods and habitat types.

We calculated correlations of species richness between phytophages, pollinators, predators, and detritivores (potential pollinators and “others” were excluded from this analysis to avoid biases, since specimens assigned to these 2 guilds were also assigned to other guilds). Correlations were observed for each sampling method at 2 levels: across all pooled sampling plots (*n* = 40) and by habitat type (*n* = 10). Pearson correlation coefficients were calculated to assess cross-taxon relationships among ecological guilds. To evaluate the statistical significance of the observed correlations, we computed *P* values using permutation-based correlation tests. Correlations with *P* < 0.05 were considered statistically significant.

## Results

### Efficiency of the Megabarcoding Protocol

A total of 15,312 brachyceran specimens were collected across the 10 sites, from which 13,877 high-quality COI sequences were recovered (success rate: 90.65%; Table 1). At a 2% p-distance threshold, sequences were clustered into 823 MOTUs, compared to 783 and 746 MOTUs at 3% and 4%, respectively (Supplementary Table S3). The strong correlation among thresholds (0.9986 to 0.9994), supported by our abundant and highly diverse molecular dataset, confirmed the robustness of the MOTU delimitation, and the 2% cut-off was used for subsequent analyses. A total of 307 MOTUs occurred as singletons, 121 as doubletons, 237 with 2 to 10 specimens, and 158 with 11 or more specimens. As removing singletons would have discarded 37% of MOTUs, we retained them in all downstream analyses and figures. For hand-net specimens, the average body length was 3.23 mm (SD: ±2.57 mm), while for pan trap samples, it was 3.11 mm (SD: ±2.09 mm). MOTUs were assigned to 48 families, confirming a broad taxonomic coverage (Supplementary Table S2). We found that 8% of barcodes had been misassigned at the family level. This corresponded to 12% of unique haplotypes (*n* = 380) and 13% of MOTUs (*n* = 104). In some cases, assignments involved taxonomically related families (eg Calliphoridae identified as Anthomyiidae), whereas in others, they involved unrelated families (eg Canacidae identified as Tachinidae).

**Table 1.** Specimen counts, MOTU richness (2% threshold), and numbers of unique and shared MOTUs by sampling method and habitat

	Specimen count		MOTUs		Unique MOTUs		Common MOTUs
Habitat type	Net	Pan traps	Net	Pan traps	Net	Pan traps	Net and Pan traps
**Mobile dunes**	1,147	1,943	149	205	69	125	77
**Fixed dunes**	604	1,973	156	184	93	121	63
**Wetland**	1,658	3,904	291	260	177	146	114
**Forest**	608	2,040	212	256	124	168	88
**Total**	4,017	9,860	563*	546*	281*	262*	282*

Totals are shown. Asterisks indicate absolute MOTU totals, de-duplicated across habitats.

### Complementarity of Pan Traps and Hand-Netting

**Fig. 2.**
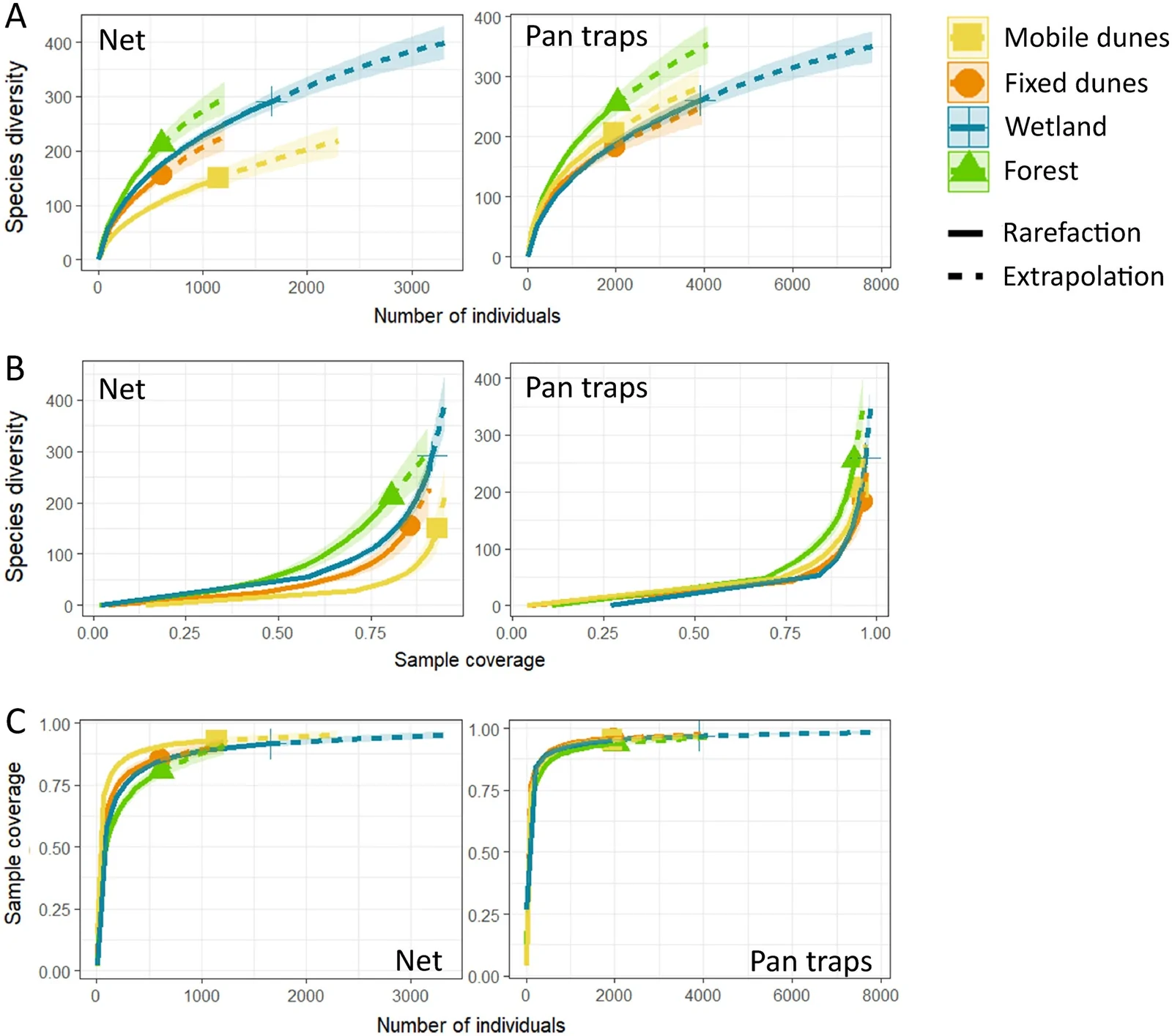
A) Sample size, B) sample completeness, and C) coverage-based rarefaction (solid line) and extrapolation (dashed line) curves comparing net and pan traps samples.

Beta diversity analysis revealed a mean compositional dissimilarity of 60.0% among assemblages from different habitats and sampling methods. Within habitats, dissimilarity between net and pan trap samples was highest in fixed dunes (64.0%) and lowest in mobile dunes (55.0%). In most habitats, turnover was the dominant component of dissimilarity, particularly in fixed dunes, where it accounted for 60.5%, suggesting that pan traps and hand nets capture distinct subsets of the local communities. By contrast, the nestedness component peaked in mobile dunes (8.3%), suggesting that in this habitat, pan traps and nets have captured partly overlapping communities. The two sampling methods differed in sample coverage (Fig. 2C; Supplementary Table S4), with pan traps generally outperforming nets. Average sample coverage across the 4 habitats was 0.966 (SD: ±0.01) for pan traps versus 0.876 (SD: ±0.06) for nets.

Hand-netting yielded 562 MOTUs (159 singletons) belonging to 45 families, whereas pan traps yielded 543 MOTUs (148 singletons) belonging to 43 families. Across all methods, 281 MOTUs (34% of the total) were unique to netting, and 262 (32%) were unique to pan traps, while only 282 MOTUs were shared (Table 1). Thus, relying on a single sampling method would have resulted in the loss of approximately one-third of the total MOTU diversity. Rarefaction and extrapolation curves did not reach an asymptote for any habitat (Fig. 2A; Supplementary Table S4). Pan traps generally recovered more MOTUs per habitat, showing overall greater consistency in surveying species richness. The only exception concerned wetland samples. In this habitat, hand nets outperformed pan traps, yielding 291 MOTUs (while 260 MOTUs were collected through pan traps). Supplementary Fig. S1 shows sample size, sample completeness, and coverage-based rarefaction curves for the full dataset (ie combining both hand-net- and pan trap-collected samples). Overall, wetlands showed the highest absolute number of MOTUs (435) and the highest number of rare MOTUs, with 111 singletons and 84 doubletons. Extrapolation curves indicate that increased sampling effort would yield the largest increase in newly detected MOTUs in wetlands and forests (Supplementary Fig. S1A; Supplementary Table S4).

**Fig. 3.**
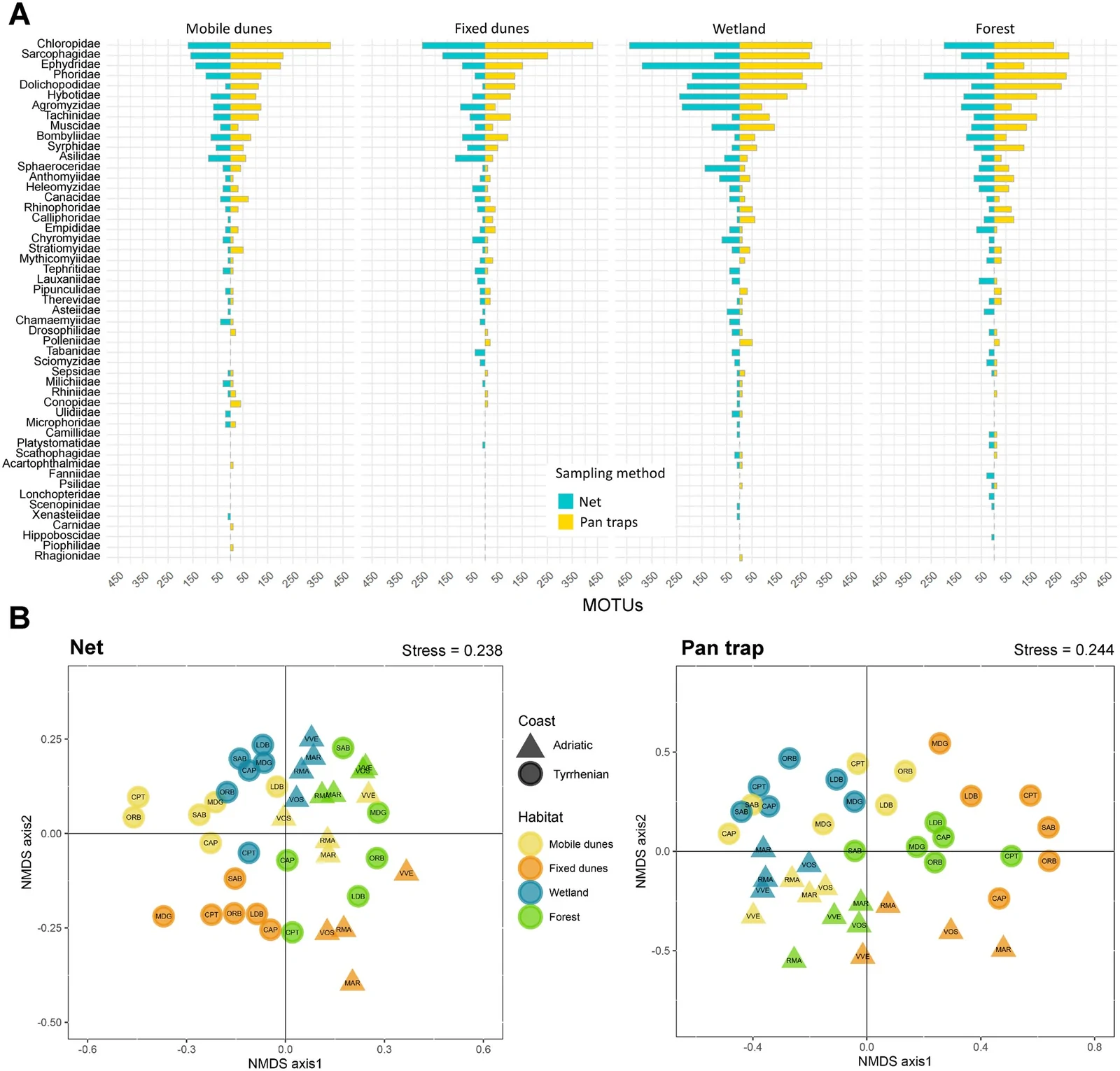
A) Forest plot showing MOTU richness across habitat types (turquoise = hand net; yellow = pan traps). Clust700 (Dolichopodidae) was excluded from visualization as it represented a strong outlier (2,362 specimens collected across the 4 habitats, 98.5% of which were captured with pan traps). B) Non-linear Multidimensional Scaling (NMDS) clustering of dipteran assemblages collected across coastal habitats using hand net (left) and pan traps (right).

Overall, nets were particularly effective in detecting certain ecological guilds, such as phytophages and predators. Family-specific patterns reflected ecological traits: Sarcophagidae and Tachinidae were mainly captured by pan traps; Dolichopodidae diversity was better represented by pan traps in both mobile and fixed dunes and forests, while Tephritidae, Asilidae, and Empididae were primarily collected by nets (Fig. 3; Supplementary Table S5). The distribution of abundances across habitats, collected using both methods is shown in Supplementary Fig. S2 and Supplementary Table S5.

The NMDS plots were supported by low stress values (net = 0.238; pan traps = 0.244). For both sampling methods, they revealed partial clustering of assemblages from mobile dunes and wetlands (Fig. 3B). Assemblages from fixed dunes were clearly separated from the others, except for a few sampling plots that are geographically close, with net-collected assemblages showing the lowest values along axis 2, and pan trap-collected assemblages showing the highest values along axis 1. Forest assemblages were more clearly separated from those of other habitats when collected using pan traps, while hand-netting yielded assemblages that partially overlapped with the other 3 habitats. The NMDS further highlighted a clear distinction between assemblages collected within sites from the Adriatic coast and sites from the Tyrrhenian coast. This separation was evident across all habitats for pan trap samples and across mobile dunes, fixed dunes, and wetlands for hand-net samples. MRM found a positive relationship between geographic distance and Bray–Curtis dissimilarity among fly assemblages, based on data from both sampling methods, yet this only partially explains compositional differences among assemblages (net: *R*^2^ = 0.008, *P* value = 0.01; pan traps: *R*^2^ = 0.02, *P* value = 0.001).

### Guild Diversity across Coastal Habitats

**Fig. 4.**
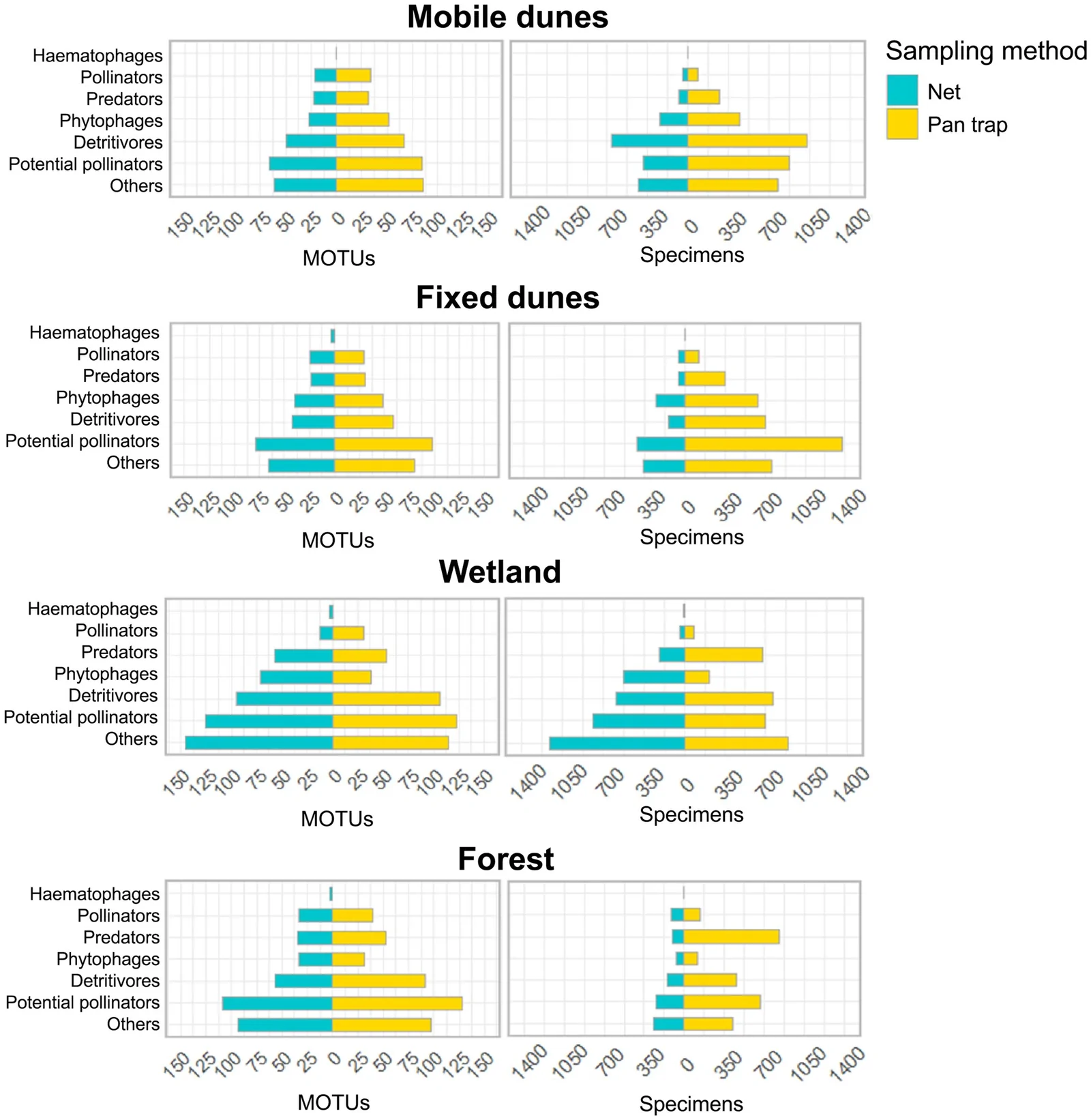
MOTU richness and specimen abundance across ecological guilds and sampling methods: A) mobile dunes, B) fixed dunes, C) wetland, D) forest; turquoise = hand net; yellow = pan traps. Several families contributed to multiple guilds; Clust700 (Dolichopodidae) was excluded from visualization.

Ecological-guild analysis revealed marked habitat-specific patterns. Although wetlands and mobile dunes showed comparable specimen/MOTU ratio (12.72 and 11.4, respectively), wetlands harbored the highest guild diversity, with a strong representation of detritivores and potential pollinators, whereas mobile dunes showed the lowest (Fig. 4; Supplementary Table S6).

Charismatic pollinators such as Syrphidae and Bombyliidae were consistently rare, while flower-visiting taxa (“potential pollinators”) (eg Chloropidae, Milichiidae, Muscidae) dominated across habitats (Table 2). Method comparison showed guild-specific patterns: nets were more effective for phytophages, pan traps for pollinators and detritivores, while predators were evenly represented across both samples. Despite these differences, the overlap between methods was substantial (27% to 40% across guilds), providing a consistent picture of ecological group representation. Cross-taxon correlation analyses revealed no consistent patterns across habitats (Supplementary Fig. S3). When analyzing pairwise correlation of ecological guilds across pooled habitat types (*n* = 40), we found positive correlations among all ecological groups for pan trap samples and a strong positive correlation between detritivores and both phytophages and predators, and between phytophages and predators (Supplementary Fig. S4).

**Table 2.** Specimen counts and MOTU richness (2% threshold) of potential pollinators (non-exclusive flower visitors) and exclusive pollinators by habitat

	Potential pollinators		Exclusive pollinators	
Habitat type	Specimen count	MOTUs	Specimen count	MOTUs
**Mobile dunes**	1261	113	131	46
**Fixed dunes**	1790	140	174	45
**Wetland**	1476	199	117	38
**Forest**	919	185	253	61
**Total**	5446	372*	675	116*

Totals are shown. Asterisks indicate absolute MOTU totals, de-duplicated across habitats.

## Discussion

Despite their ecological importance and extraordinary richness, Diptera remain among the most neglected groups in biodiversity research, with few exceptions of charismatic and/or economically relevant families (eg Syrphidae or Tephritidae). We present the first large-scale survey of fly diversity in Mediterranean coastal habitats, testing 2 complementary sampling techniques combined with a high-throughput DNA barcoding protocol. Our approach recovered 13,877 high-quality COI sequences, clustered into 823 MOTUs across 48 families, revealing an unexpectedly high diversity given that coastal habitats are generally regarded as species-poor due to their extreme environmental conditions and strong anthropogenic pressures. The large number of individuals analyzed, spanning numerous families, provided robust support for testing correlations in MOTU counts across distance thresholds (0.9986 to 0.9994). By contrast, restricting sampling to a few (charismatic) families would have reduced statistical power. This richness, amounting to more than 10% of all dipteran species so far reported from Italy (Pape et al. 2015), highlights the effectiveness of our protocol in capturing a substantial fraction of the group’s diversity.

The high sequencing success rate (>90%) and the ability to validate family-level identifications with limited expert input further demonstrate the efficiency of this approach, critical for large-scale monitoring, time-sensitive biodiversity surveys, and especially for dark taxa. Eight percent of barcodes were incorrectly assigned to families by BLAST, reflecting taxonomic errors, contamination issues, and the limited representation of many taxa in reference repositories (Piemontese et al. 2020, Recuero et al. 2024). Accurate species assignment of query sequences depends on the taxonomic coverage of reference repositories (Ekrem et al. 2007, Kvist 2013). However, hyperdiverse groups are disproportionately affected by incomplete representation of intra- and interspecific diversity, which increases the risk of incorrect matches, misidentifications, and taxonomic ambiguity (Meier et al. 2006, Virgilio et al. 2012, Pentinsaari et al. 2020). In this context, morphological validation of 1 specimen per MOTU ensured reliable family-level identifications, overcoming limitations of reference repositories while minimizing expert taxonomic effort. Importantly, accurate family-level data on fly diversity were obtained without the need for expert taxonomists. BLAST suggestions accelerated the identification process, while the simplified inspection protocol facilitated family-level assignments. This reverse approach greatly reduced processing time, enabling the rapid handling of large sample volumes, finally enabling a broad-scale biodiversity assessment. Specimens were clustered into MOTUs and retained intact for subsequent expert-based morphological examination at the species-level, enabling integrative taxonomy (eg Hartop et al. 2022) and facilitating species discovery within Diptera (see Cerretti et al. 2026). By contrast, hand-netting is commonly conducted as non-lethal sampling (Pollard and Yates 1993, Lecq et al. 2015), which often permits only higher-level identifications, especially for small-bodied specimens (O’Connor et al. 2019), and needs to be supported by additional monitoring methods (Lortie et al. 2012), thereby limiting downstream analyses of taxonomic and functional diversity.

### Comparing 2 Sampling Methods: Hand-Netting and Pan Trapping

We combined 2 complementary sampling methods and quantified the overlap in MOTUs captured. While transect sampling, active hand-netting, sweeping, and targeted collecting are sensitive to operator variability (Sutherland et al. 2015, O’Connor et al. 2019), pan traps are a passive, low-cost method that does not require specific expertise, though their effectiveness still depends on appropriate placement. We used yellow pan traps, widely recommended for Diptera (Grootaert et al. 2010, Dirrigl 2012) and effective at capturing diverse insect assemblages, including phytophages (Kirk 1984) and predators (Leksono et al. 2005).

Hand-netting and pan trapping can yield samples that differ in number and distribution of individuals (Westphal et al. 2008, but see O’Connor et al. 2019), in species counts (Grundel et al. 2011, Popic et al. 2013), and in trait composition (McCravy et al. 2019). Hand-netting tends to favor conspicuous, larger, and often common species and typically yields fewer individuals than traps (Dennis et al. 2006, O’Connor et al. 2019, Thompson et al. 2021). Pan traps, by contrast, allow standardized collection, often capture more specimens (Grundel et al. 2011, Thompson et al. 2021), smaller in body size (larger insects can more readily escape [Cane et al. 2000]), and can return higher species counts (Westphal et al. 2008, O’Connor et al. 2019). Based on the measurements of 96% of our MOTUs, we were able to observe that mean body size of collected brachycerans was slightly larger in hand-net samples (3.23 mm, SD: ±2.57) than in pan trap samples (3.11 mm, SD: ±2.09).

Considering the broad taxonomic and functional scope of our sampling, these outcomes differ from much of the literature, largely focused on pollinator communities (especially hoverflies and bees). Only 14% of MOTUs (5% of specimens) represented large-bodied, charismatic pollinators (eg Syrphidae, Bombyliidae, Tachinidae), with hand-netting yielding 62 MOTUs and pan trapping 85. In contrast, potential pollinators from small-bodied dipteran families (eg Chloropidae, Milichiidae) accounted for 45% of MOTUs (39% of specimens): hand-netting yielded 253 MOTUs, while pan trapping yielded 248 MOTUs. This strong disparity indicates that small-bodied taxa likely constitute a substantial portion of coastal potential pollinators, and that dipteran contributions to dune pollination may be overall underestimated relative to large-bodied Hymenoptera-dominated networks described in the literature.

Overall, the number of MOTUs was broadly comparable across sampling methods (562 vs. 543), and the number of MOTUs exclusively collected by each method was similar: without hand-netting, 34% of MOTUs (281) would have been overlooked, while without pan traps, 32% (262) would not have been detected (Table 1). In contrast, the number of specimens collected by hand-netting and pan trapping differed markedly (4,018 vs. 9,962, respectively). Through our approach, a high number of detritivorous and phytophagous individuals were collected, finally increasing the total number of specimens. Differences in specimen numbers likely reflect differences in sampling effort and collection time (Rhoades et al. 2017). Sampling efforts cannot be fully standardized across the 2 methods due to intrinsic characteristics, while the combination of the 2 can help mitigate method-specific gaps (Le Féon et al. 2016). Pan traps operated for 8 h, and active hand-netting was always supported by continuous plant sweeping during the 30-min collection sessions. Furthermore, sampling within plots was conducted by a non-expert taxonomist; this may have reduced taxonomic “cherry-picking” and yielded abundant and diverse hand-net catches.

Combining pan traps and hand-netting increased coverage and revealed complementary patterns, since the 2 methods captured different dipteran taxa with distinct ecological traits. Sarcophagidae and Tachinidae were mostly captured by pan traps, consistent with their flower-visiting habits; Dolichopodidae diversity was better represented by pan traps in dunes and forests, which could be attributable to their foliage-seeking behavior as predators (Ekbom 1980, Kirk 1984); Asilidae were primarily collected by hand-netting, likely reflecting their limited attraction to visual cues targeted by pan traps and their perch-hunting behaviors on the ground, branches, and trunks, which reduces encounter rates with traps. Overall, this is consistent with previous studies, confirming that different insect families are disproportionately represented depending on the method used (Missa et al. 2009, O’Connor et al. 2019, Hutchinson et al. 2022), and that all sampling approaches carry inherent biases (Borkent et al. 2018). This is particularly relevant in heterogeneous habitats like coastal dunes, where insect assemblages can vary considerably across microhabitats and temporal scales.

Between 27 and 40% of MOTUs, depending on guild, were shared between the 2 sampling methods. The number of specimens and MOTUs assigned to pollinators and potential pollinator guilds in mobile dunes, fixed dunes, and forest communities was higher for pan trap-collected assemblages. The first 2 habitats featured limited, patchy floral resources; several forest sites likewise showed low flower availability, with some species occurring mainly in ecotone areas. Both hand-netting and pan trap sampling are influenced by flower availability and density within the sampled habitat (O’Connor et al. 2019, Westerberg et al. 2021, Chamorro et al. 2023), although it is difficult to establish a general pattern of this correlation (Cane et al. 2000, Wood et al. 2015). The attractivity of pan traps in conditions where flower patches are poorly abundant could explain the high number of specimens collected within the 3 habitats mentioned above. In both mobile and fixed dunes, pan traps outperformed hand-netting even for detritivores, predators, and phytophages; in forests, higher counts were observed for detritivores and predators. This pattern indicates that pan traps effectively sample a broad spectrum of trophic guilds, as a diversity of flies is attracted to visual cues, with predator captures further enhanced by their spatial coupling with prey. For wetlands, we observed different patterns: hand-netting delivered a high number of phytophages, while pan traps performed better at collecting predators; roughly, a similar number of detritivores were collected through the 2 methods. In contrast, no significant differences were observed for potential pollinator abundance. Importantly, our plots aimed to include all representative microhabitats available within each of the 4 habitats. In biodiversity censuses, covering a range of habitats and microhabitats within a plot is recommended to avoid missing important taxonomic and functional groups. Special attention should be given to the choice of sampling scheme to ensure alignment with research objectives, as intrinsic characteristics of the systems can influence the suitability of methods (Gullan and Cranston 2010, Prado et al. 2017), and sampling methods should be adjusted to functional traits of the target group (Carrié et al. 2017, Prendergast and Hogendoorn 2021, Thompson et al. 2021).

The NMDS plots based on pan trap samples showed stronger differentiation than those based on net samples and revealed a clear separation between assemblages from mobile and fixed dunes. This indicates that, despite the limited spatial distance between these habitat types, microhabitat differences are sufficient to support distinct communities, a pattern consistently recovered by both sampling methods (Fig. 3B). Both habitats may therefore be particularly relevant for potential pollinators. Pan traps revealed a clear biogeographic pattern, separating Tyrrhenian from Adriatic coastal assemblages regardless of habitat type, likely reflecting variation in climatic conditions and plant diversity between the 2 Italian regions. In contrast, sweep-net sampling produced a different pattern, in which this separation disappeared for forest habitats. This discrepancy may reflect the lower efficiency of sweep-netting in forests with a sparse understory vegetation layer, where this method may have mainly captured accessible, generalist species shared across different habitat types while underrepresenting forest-specialist or habitat-specific taxa. Resolving assemblages to species level will be essential to identify the taxa and ecological guilds structuring each coast, thereby informing conservation strategies in highly exploited coastal systems. Overall, wetlands supported the highest taxonomic and functional diversity, dominated by detritivores and potential pollinators, whereas mobile dunes exhibited the lowest. Despite the use of 2 complementary sampling methods, species-accumulation curves did not reach an asymptote in any habitat, suggesting that increased sampling efforts or additional sampling techniques would likely be required to narrow remaining taxonomic gaps if estimating total species richness is the goal. Malaise traps could be deployed for this purpose; however, their conspicuousness in high-tourist areas makes them vulnerable to vandalism, and strong winds may damage or dislodge them.

Combining hand-netting and plant sweeping with pan traps is recommended to increase sampling efficacy across different ecological roles, including taxa with parasitoid life cycles (Mazon and Bordera 2008) as well as adult pollinators (Cane et al. 2000, Stephen and Rao 2007, Westphal et al. 2008, Montgomery et al. 2021). In the EU PoMS framework, pan traps are indeed considered a complementary module for collecting “other insects” (Potts et al. 2024). Consistent with previous studies (Shweta and Rajmohana 2018), the joint use of these methods proved effective for species discovery, allowing the detection of dominant guilds in sandy coastal habitats that transects or pan traps alone might have failed to capture. By jointly analyzing taxonomic and guild diversity across methods, community composition can be assessed more robustly, and monitoring protocols can be designed to minimize sampling bias (Chao et al. 2021, Larson et al. 2021).

## Conclusions

Available evaluations of sampling efficiency largely focus on pollinators, whereas other ecological guilds are seldom assessed, particularly in large-scale, multi-taxon frameworks. Hyperdiverse groups are typically accessed through targeted sampling or opportunistic subsampling from large Malaise-trap catches, primarily within a taxonomic context. In contrast, comprehensive and standardized syntheses of multi-taxon sampling efficiency that include dark taxa remain scarce. Our methodological approach, combining cost-efficient sampling with specimen processing through a reverse approach, highlights the value of high-throughput, multi-method workflows for documenting hyperdiverse yet neglected insect communities. Our protocol further enabled high taxonomic-level ecological-guild analyses that revealed overlooked roles of dipterans in coastal ecosystems, particularly as pollinators and detritivores. More broadly, our findings underscore the need for standardized, scalable, and shared protocols to survey the neglected fraction of biodiversity. Such approaches will provide robust baselines to help overcome the taxonomic impediment and ensure that insect taxa are adequately represented in conservation strategies, especially in fragile systems like coastal dunes.

## Supplementary Material

saag025_Supplementary_Data

## Data Availability

The main dataset, specimen images, supplementary materials, and the FASTA file generated during this study are publicly available at https://doi.org/10.5281/zenodo.17590749.

## References

[saag025-B1] Ascenzi A , Marini L, Geppert C, et al 2025. Data from: beyond conventional pollinators: advancing diversity assessments of neglected insect taxa [data set]. Zenodo Digital Repository. 10.5281/zenodo.17590749.

[saag025-B2] Awad J , Brar G, Cadwalader E, et al 2025. Defining the decline: a glossary relevant to insect decline. J. Insect Sci. 25:4. 10.1093/jisesa/ieaf048. PMC12070476 40358519

[saag025-B3] Barkalov AV , Mutin VA. 2019. Checklist of the hover-flies (Diptera, Syrphidae) of Russia. EEJ. 17:466–512. 10.15298/euroasentj.17.6.12

[saag025-B4] Baselga A , Orme CDL. 2012. betapart: an R package for the study of beta diversity. Methods Ecol. Evol. 3:808–812. 10.1111/j.2041-210X.2012.00224.x

[saag025-B5] Biswas S , Rajkonwar J, Jena SR, et al 2025. Detection of a sympatric cryptic species mimicking *Aedes albopictus* (Diptera: Culicidae) in dengue and chikungunya endemic forest villages of Tripura, India, posing a daunting challenge for vector research. Sci. Rep. 15:14237. 10.1038/s41598-025-96146-9 40274861 PMC12022023

[saag025-B6] Borkent A , Brown BV, Adler PH, et al 2018. Remarkable fly (Diptera) diversity in a patch of Costa Rican cloud forest: why inventory is a vital science. Zootaxa 4402:53–90. 10.11646/zootaxa.4402.1.3 29690278

[saag025-B7] Breeze TD , Bailey AP, Balcombe KG, et al 2021. Pollinator monitoring more than pays for itself. J. Appl. Ecol. 58:44–57. 10.1111/1365-2664.13755

[saag025-B8] Cane JH , Minckley RL, Kervin LJ. 2000. Sampling bees (Hymenoptera: Apiformes) for pollinator community studies: pitfalls of pan-trapping. J Kans Entomol Soc 73:225–231.

[saag025-B9] Cardoso P , Leather SR. 2019. Predicting a global insect apocalypse. Insect Conserv. Diversity 12:263–267. 10.1111/icad.12367

[saag025-B10] Carrié R , Andrieu E, Cunningham SA, et al 2017. Relationships among ecological traits of wild bee communities along gradients of habitat amount and fragmentation. Ecography 40:85–97. 10.1111/ecog.02632

[saag025-B11] Caruso V , Hartop E, Chimeno C, et al 2024. An integrative framework for dark taxa biodiversity assessment at scale: a case study using *Megaselia* (Diptera, Phoridae). Insect Conserv. Diversity 17:968–987. 10.1111/icad.12762.

[saag025-B12] Cerretti P , Nania D, Di Marco M, et al 2026. Declining rates of species description call for improved taxonomic strategies: insights from a megadiverse insect order. Syst. Entomol. 51:e70019. 10.1111/syen.70019

[saag025-B13] Chacoff NP , Vázquez DP, Lomáscolo SB, et al 2012. Evaluating sampling completeness in a desert plant–pollinator network. J. Anim. Ecol. 81:190–200. 10.1111/j.1365-2656.2011.01883.x. 21815890

[saag025-B14] Chamorro FJ , Faria CMA, Araújo FS, et al 2023. Elevated pan traps optimise the sampling of bees, including when the availability of floral resources is high. Insect Conserv. Diversity 16:16–32. 10.1111/icad.12621

[saag025-B15] Chao A , Henderson PA, Chiu C-H, et al 2021. Measuring temporal change in alpha diversity: a framework integrating taxonomic, phylogenetic and functional diversity and the iNEXT.3D standardization. Methods Ecol. Evol. 12:1926–1940. 10.1111/2041-210X.13682.

[saag025-B16] Chao A , Jost L. 2012. Coverage-based rarefaction and extrapolation: standardizing samples by completeness rather than size. Ecology 93:2533–2547. 10.1890/11-1952.1 23431585

[saag025-B17] Chimeno C , Hausmann A, Schmidt S, et al 2022. Peering into the darkness: DNA barcoding reveals surprisingly high diversity of unknown species of Diptera (Insecta) in Germany. Insects 13:82. 10.3390/insects13010082 35055925 PMC8779287

[saag025-B18] Chimeno C , Rulik B, Manfrin A, et al 2023. Facing the infinity: tackling large samples of challenging Chironomidae (Diptera) with an integrative approach. PeerJ 11:e15336. 10.7717/peerj.15336 37250705 PMC10211366

[saag025-B19] D’Souza ML , Van der Bank M, Shongwe Z, et al 2021. Biodiversity baselines: tracking insects in Kruger National Park with DNA barcodes. Biol. Conserv. 256:109034. 10.1016/j.biocon.2021.109034

[saag025-B20] Dennis RLH , Shreeve TG, Isaac NJB, et al 2006. The effects of visual apparency on bias in butterfly recording and monitoring. Biol. Conserv. 128:486–492. 10.1016/j.biocon.2005.10.015

[saag025-B21] Dirrigl FJ. Jr. 2012. Effectiveness of pan trapping as a rapid bioinventory method of freshwater shoreline insects of subtropical texas. Southwest. Entomol. 37:133–139. 10.3958/059.037.0205

[saag025-B22] Disney HL , Erzinclioglu YZ, de C, et al 1982. Collecting methods and the adequacy of attempted fauna surveys, with reference to the Diptera. Field Studies 5:607–621.

[saag025-B23] Dousti AF , Hayat R. 2006. A catalogue of the Syrphidae (Insecta: Diptera) of Iran. J. Entomol. Res. Soc. 8:5–38.

[saag025-B24] Ekbom B. 1980. Use of traps for detection of whitefly attack and a note on the colour preference of *Encarsia formosa*. Vartskyddnotiser 44:115–120.

[saag025-B25] Ekrem T , Willassen E, Stur E. 2007. A comprehensive DNA sequence library is essential for identification with DNA barcodes. Mol. Phylogenet. Evol. 43:530–542. 10.1016/j.ympev.2006.11.021 17208018

[saag025-B26] Elbrecht V , Braukmann TWA, Ivanova NV, et al 2019. Validation of COI metabarcoding primers for terrestrial arthropods. PeerJ 7:e7745. 10.7717/peerj.7745 31608170 PMC6786254

[saag025-B27] Elbrecht V , Leese F. 2017. Validation and development of COI metabarcoding primers for freshwater macroinvertebrate bioassessment. *Front Environ Sci*. 5. 10.3389/fenvs.2017.00011

[saag025-B28] EU Habitats Directive. 1992. Council Directive 92/43/EEC of 21 May 1992 on the conservation of natural habitats and wild fauna and flora. Off J Eur Comm 206:7–50.

[saag025-B29] European Commission. 2023. Horizon Europe Work Programme 2023-2025—9. Food, bioeconomy, natural resources, agriculture and environment: European Commission web page. https://ec.europa.eu/info/funding-tenders/opportunities/docs/2021-2027/horizon/wp-call/2025/wp-9-food-bioeconomy-natural-resources-agriculture-and-environment_horizon-2025_en.pdf

[saag025-B32] Favret C , Lessard V, Trépanier A, et al 2019. Voegtlin-style suction traps measure insect diversity and community heterogeneity. Insect Conserv. Diversity 12:373–381. 10.1111/icad.12368

[saag025-B33] Geiger M , Morinière J, Hausmann A, et al 2016. Testing the Global Malaise Trap program - how well does the current barcode reference library identify flying insects in Germany? Biodivers. Data J. 4:e10671. 10.3897/bdj.4.e10671 PMC5136679 27932930

[saag025-B35] Germann C , Pollet M, Wimmer C, et al 2011. Molecular data sheds light on the classification of long-legged flies (Diptera: Dolichopodidae). Invertebrate Systematics 25:303–321. 10.1071/IS11029

[saag025-B36] Goslee SC , Urban DL. 2007. The ecodist package for dissimilarity-based analysis of ecological data. J. Stat. Soft. 22:1–19. 10.18637/jss.v022.i07

[saag025-B37] Grange MC , Munoz F, Moretti M, et al 2021. Designing sampling protocols for plant-pollinator interactions—timing, meteorology, flowering variations and failed captures matter. Bot. Lett. 168:324–332. 10.1080/23818107.2021.1964596

[saag025-B38] Grootaert P , Pollet M, Dekoninck W, et al 2010. Sampling insects: general techniques, strategies and remarks. In: Eymann J, editor. Manual on field recording techniques and protocols for all taxa biodiversity inventories and monitoring. Abc Taxa. p. 337–399.

[saag025-B39] Grundel R , Frohnapple KJ, Jean RP, et al 2011. Effectiveness of bowl trapping and netting for inventory of a bee community. Env. Entom. 40:374–380. 10.1603/EN09278

[saag025-B40] Gullan J , Cranston P. 2010. The insects: an outline of entomology. WileyBlackwell. p. 624.

[saag025-B41] Habel JC , Gossner MM, Schmitt T. 2021. Just beautiful?! What determines butterfly species for nature conservation. Biodivers. Conserv. 30:2481–2493. 10.1007/s10531-021-02204-9

[saag025-B42] Habel JC , Ulrich W, Biburger N, et al 2019. Agricultural intensification drives butterfly decline. Insect Conserv. Diversity 12:289–295. 10.1111/icad.12343

[saag025-B43] Hartop E , Lee L, Srivathsan A, et al 2024. Resolving biology’s dark matter: species richness, spatiotemporal distribution, and community composition of a dark taxon. BMC Biol. 22:215. 10.1186/s12915-024-02010-z 39334308 PMC11438253

[saag025-B44] Hartop E , Srivathsan A, Ronquist F, et al 2022. Towards large-scale integrative taxonomy (LIT): resolving the data conundrum for dark taxa. Syst. Biol. 71:1404–1422. 10.1093/sysbio/syac033 35556139 PMC9558837

[saag025-B45] Hausmann A , Krogmann L, Peters R, et al 2020. GBOL III: DARK TAXA. barbull. 10:4. 10.21083/ibol.v10i1.6242

[saag025-B46] Hebert PDN , Floyd R, Jafarpour S, et al 2025. Barcode 100K specimens: in a single nanopore run. Mol. Ecol. Resour. 25:e14028. 10.1111/1755-0998.14028 39387679 PMC11646297

[saag025-B47] Hebert PDN , Ratnasingham S, Zakharov EV, et al 2016. Counting animal species with DNA barcodes: Canadian insects. Philos. Trans. R Soc. Lond. B Biol. Sci. 371:10. 10.1098/rstb.2015.0333 PMC4971185 27481785

[saag025-B48] Hsieh TC , Ma KH, Chao A. 2016. iNEXT: an R package for rarefaction and extrapolation of species diversity (Hill numbers). Methods Ecol. Evol. 7:1451–1456. 10.1111/2041-210X.12613

[saag025-B49] Hutcheson J. 1990. Characterization of terrestrial insect communities using quantified, Malaise-trapped Coleoptera. Ecol. Entomol. 15:143–151. 10.1111/j.1365-2311.1990.tb00795.x

[saag025-B50] Hutchinson LA , Oliver TH, Breeze TD, et al 2022. Inventorying and monitoring crop pollinating bees: evaluating the effectiveness of common sampling methods. Insect Conserv. Diversity 15:299–311. 10.1111/icad.12557

[saag025-B51] Irvine KM , Woods SA. 2007. Evaluating shading bias in Malaise and window-pane traps. J. Acad. Entomol. Soc. 3:38–48.

[saag025-B52] Karlsson D , Hartop E, Forshage M, et al 2020. The Swedish Malaise Trap Project: a 15 year retrospective on a countrywide insect inventory. Biodivers. Data J. 8:e47255. 10.3897/BDJ.8.e47255 32015667 PMC6987249

[saag025-B53] Katoh K , Standley DM. 2013. MAFFT multiple sequence alignment software version 7: improvements in performance and usability. Mol. Biol. Evol. 30:772–780. 10.1093/molbev/mst010 23329690 PMC3603318

[saag025-B54] Katoh TK , Ting C-T, Tsaur S-C, et al 2025. Discovery and descriptions of three cryptic species of the genus *Drosophila* Fallén (Diptera: Drosophilidae) from laboratory-maintained strains. Zootaxa 5661:573–587. 10.11646/zootaxa.5661.4.8. 41119813

[saag025-B55] Kirk WDJ. 1984. Ecologically selective coloured traps. Ecol. Entomol. 9:35–41.

[saag025-B56] Kvist S. 2013. Barcoding in the dark? A critical view of the sufficiency of zoological DNA barcoding databases and a plea for broader integration of taxonomic knowledge. Mol. Phylogenet. Evol. 69:39–45. 10.1016/j.ympev.2013.05.012 23721749

[saag025-B57] Larson E , Poff N, Funk W, et al 2021. A unifying framework for analyzing temporal changes in functional and taxonomic diversity along disturbance gradients. Ecology 102:e03503. 10.1002/ecy.3503 34314030

[saag025-B58] Le Féon V , Henry M, Guilbaud L, et al 2016. An expert-assisted citizen science program involving agricultural high schools provides national patterns on bee species assemblages. J. Insect Conserv. 20:905–918. 10.1007/s10841-016-9927-1

[saag025-B59] Leather SR editors. 2008. Insect sampling in forest ecosystems. John Wiley & Sons.

[saag025-B60] Lecq S , Loisel A, Bonnet X. 2015. Non-lethal rapid biodiversity assessment. Ecol. Indic. 58:216–224. 10.1016/j.ecolind.2015.06.004

[saag025-B61] Leksono AS , Takada K, Koji S, et al 2005. Vertical and seasonal distribution of flying beetles in a suburban temperate deciduous forest collected by water pan trap. Insect Sci. 12:199–206. 10.1111/j.1005-295X.2005.00025.x

[saag025-B62] Leong JM , Thorp RW. 1999. Colour-coded sampling: the pan colour preferences of oligolectic and nonoligolectic bees associated with a vernal pool plant. Ecol. Entomol. 24:329–335. 10.1046/j.1365-2311.1999.00196.x

[saag025-B63] Lortie CJ , Budden A, Reid A. 2012. From birds to bees: applying video observation techniques to invertebrate pollinators. J. Poll. Ecol. 6 (January). 10.26786/1920-7603(2011)20

[saag025-B64] Marín-Armijos D , Quezada-Ríos N, Soto-Armijos C, et al 2017. Checklist of the flower flies of Ecuador (Diptera, Syrphidae). Zookeys. 691:163–199. 10.3897/zookeys.691.13328 PMC5672696 29200924

[saag025-B65] Marshall SA. 2012. Flies: the natural history and diversity of diptera. Firefly Books.

[saag025-B66] Mason F , Cerretti P, Nardi G, et al 2006. Aspects of biological diversity in the CONECOFOR plots. IV. The Invertebrate Biodiv pilot project. Annali Dell’Istituto Sperimentale per la Selvicoltura 30:51–70.

[saag025-B67] Mazon M , Bordera S. 2008. Effectiveness of two sampling methods used for collecting Ichneumonidae (Hymenoptera) in the Cabaneros National Park (Spain). Eur. J. Entomol. 105:879–888. 10.14411/eje.2008.116

[saag025-B68] McCravy KW , Geroff RK, Gibbs J. 2019. Bee (Hymenoptera: Apoidea: Anthophila) functional traits in relation to sampling methodology in a restored tallgrass prairie. Fla. Entomol. 102:134–140. 10.1653/024.102.0122

[saag025-B69] Meglécz E. 2023. COInr and mkCOInr: building and customizing a nonredundant barcoding reference database from BOLD and NCBI using a semi-automated pipeline. Mol. Ecol. Resour. 23:933–945. 10.1111/1755-0998.13756 36656075

[saag025-B70] Meier R , Blaimer BB, Buenaventura E, et al 2022. A re-analysis of the data in Sharkey et al.’s (2021) minimalist revision reveals that BINs do not deserve names, but BOLD Systems needs a stronger commitment to open science. Cladistics 38:264–275. 10.1111/cla.12407 34487362

[saag025-B71] Meier R , Hartop E, Pylatiuk C, et al 2024. Towards holistic insect monitoring: species discovery, description, identification and traits for all insects. Philos. Trans. R Soc. Lond. B Biol. Sci. 379:20230120. 10.1098/rstb.2023.0120 38705187 PMC11070263

[saag025-B72] Meier R , Kwong S, Vaidya G, et al 2006. DNA barcoding and taxonomy in Diptera: a tale of high intraspecific variability and low identification success. Syst. Biol. 55:715–728. 10.1080/10635150600969864 17060194

[saag025-B73] Meier R , Lawniczak MK, Srivathsan A. 2025. Illuminating entomological dark matter with DNA barcodes in an era of insect decline, deep learning, and genomics. Annu. Rev. Entomol. 70:185–204. 10.1146/annurev-ento-040124-014001 39353093

[saag025-B74] Meier R , Wong W, Srivathsan A, et al 2016. $1 DNA barcodes for reconstructing complex phenomes and finding rare species in specimen-rich samples. Cladistics 32:100–110. 10.1111/cla.12115 34732017

[saag025-B75] Missa O , Basset Y, Alonso A, et al 2009. Monitoring arthropods in a tropical landscape: relative effects of sampling methods and habitat types on trap catches. J. Insect Conserv. 13:103–118. 10.1007/s10841-007-9130-5

[saag025-B76] Montgomery GA , Belitz MW, Guralnick RP, et al 2021. Standards and best practices for monitoring and benchmarking insects. Front. Ecol. Evol. 8:579193. 10.3389/fevo.2020.579193

[saag025-B77] Montoya A , Pérez S, Wolff M. 2012. The diversity of flower flies (Diptera: Syrphidae) in Colombia and their neotropical distribution. Neotrop. Entomol. 41:46–56. 10.1007/s13744-012-0018-z 23950009

[saag025-B78] National Center for Biotechnology Information (NCBI). 1988. National Center for Biotechnology Information. National Library of Medicine. https://www.ncbi.nlm.nih.gov/

[saag025-B79] O’Connor RS , Kunin WE, Garratt MPD, et al 2019. Monitoring insect pollinators and flower visitation: the effectiveness and feasibility of different survey methods. Methods Ecol. Evol. 10:2129–2140. 10.1111/2041-210X.13292

[saag025-B80] Oksanen J , Simpson G, Blanchet F., et al 2022. Vegan: community Ecology Package. R package version 2.6-4. 10.32614/CRAN.package.vegan

[saag025-B81] Oosterbroek P. 1998. The European families of the Diptera: identification, diagnosis, biology. KNNV Publishing.

[saag025-B82] Orford KA , Vaughan IP, Memmott J. 2015. The forgotten flies: the importance of non-syrphid Diptera as pollinators. Proc. Biol. Sci. 282:20142934. 10.1098/rspb.2014.2934 25808886 PMC4389612

[saag025-B83] Page RDM. 2016. DNA barcoding and taxonomy: dark taxa and dark texts. Philos. Trans. R Soc. Lond. B Biol. Sci. 371:20150334. 10.1098/rstb.2015.0334 27481786 PMC4971186

[saag025-B84] Pape T , Beuk P, Pont AC, et al 2015. Fauna Europaea: Diptera—Brachycera. Biodivers. Data J. 20:e4187. 10.3897/BDJ.3.e4187 PMC4339814 25733962

[saag025-B85] Papp L , Darvas B, editors. 1997–2000. Manual of palaearctic diptera. Vol. 1–4. Science Herald.

[saag025-B86] Pentinsaari M , Blagoe GA, Hogg ID, et al 2020. A DNA barcoding survey of an Arctic arthropod community: implications for future monitoring. Insects 11:46. 10.3390/insects11010046 31936447 PMC7023425

[saag025-B87] Piemontese L , Giovannini I, Guidetti R, et al 2020. The species identification problem in mirids (Hemiptera: Heteroptera) highlighted by DNA barcoding and species delimitation studies. Eur. J. Zoo. 87:310–324. 10.1080/24750263.2020.1773948

[saag025-B88] Pollard E , Yates TJ. 1993. Monitoring butterflies for ecology and conservation: the British butterfly monitoring scheme. Springer Netherlands.

[saag025-B90] Popic TJ , Davila YC, Wardle GM. 2013. Evaluation of common methods for sampling invertebrate pollinator assemblages: net sampling out-perform pan traps. PLoS One. 8:e66665. 10.1371/journal.pone.0066665 23799127 PMC3684574

[saag025-B91] Potts SG , Bartomeus I, Biesmeijer K, et al; European Commission, Joint Research Centre. 2024. Refined proposal for an EU Pollinator Monitoring Scheme. JRC138660. Publications Office of the European Union. https://data.europa.eu/doi/10.2760/2005545.

[saag025-B92] Prado SG , Ngo HT, Florez JA, et al 2017. Sampling bees in tropical forests and agroecosystems: a review. J. Insect Conserv. 21:753–770. 10.1007/s10841-017-0018-8

[saag025-B93] Prendergast KS , Hogendoorn K. 2021. FORUM: methodological shortcomings and lack of taxonomic effort beleaguer Australian bee studies. Austral Ecol. 46:880–884. 10.1111/aec.12998

[saag025-B94] R Core Team. 2022. R: a language and environment for statistical computing [Computer software]. R Foundation for Statistical Computing. https://www.r-project.org/

[saag025-B95] Ratnasingham S , Hebert PDN. 2007. BOLD: the barcode of life data system (www.barcodinglife.org). Mol. Ecol. Notes. 7:355–364. 10.1111/j.1471-8286.2007.01678.x 18784790 PMC1890991

[saag025-B96] Ratnasingham S , Hebert PDN. 2013. A DNA-based registry for all animal species: the Barcode Index Number (BIN) system. PLoS One. 8:e66213. 10.1371/journal.pone.0066213 23861743 PMC3704603

[saag025-B97] Recuero E , Etzler FE, Caterino MS, et al 2024. Most soil and litter arthropods are unidentifiable based on current DNA barcode reference libraries. Curr. Zool. 70:637–646. 10.1093/cz/zoad051 39463700 PMC11502157

[saag025-B98] Rhoades P , Griswold T, Waits L, et al 2017. Sampling technique affects detection of habitat factors influencing wild bee communities. J. Insect Conserv. 21:703–714. 10.1007/s10841-017-0013-0

[saag025-B99] Riccardi PR , Hartop E. 2024. Large-scale integrative taxonomy of Swedish grass flies (Diptera, Chloropidae) reveals hitherto unknown complexity of a dark taxon. Zoologica Scripta 53:614–631. 10.1111/zsc.12663

[saag025-B100] Riedel A , Sagata K, Suhardjono YR, et al 2013. Integrative taxonomy on the fast track—towards ore sustainability in biodiversity research. Front. Zool. 10:15. 10.1186/1742-9994-10-15 23537182 PMC3626550

[saag025-B101] Rodrigues BL , Galati E. 2023. Molecular taxonomy of phlebotomine sand flies (Diptera, Psychodidae) with emphasis on DNA barcoding: a review. Acta Trop. 238:106778. 10.1016/j.actatropica.2022.106778 36435214

[saag025-B102] Sánchez-Fernández D , Fox R, Dennis RLH, et al 2021. How complete are insect inventories? An assessment of the British butterfly database highlighting the influence of dynamic distribution shifts on sampling completeness. Biodivers. Conserv. 30:889–902. 10.1007/s10531-021-02122-w

[saag025-B104] Shirali H , Ascenzi A, Wührl L, et al 2026. InsectMorphoAI: A deep learning framework for automated insect morphometrics and biomass estimation with taxon-specific volumetric validation. *Ecol Inform*, 103854. 10.1016/j.ecoinf.2026.103854

[saag025-B105] Shweta M , Rajmohana K. 2016. A comparison of efficiencies of sweep net, yellow pan trap and Malaise trap in sampling Platygastridae (Hymenoptera: Insecta). J. Exp. Zool. 19:393–396.

[saag025-B106] Shweta M , Rajmohana K. 2018. A comparison of sweep net, yellow pan trap and malaise trap for sampling parasitic Hymenoptera in a backyard habitat in Kerala. Entomon 43:33–44.

[saag025-B107] Smith TJ , Mayfield MM. 2015. Diptera species and functional diversity across tropical Australian countryside landscapes. Biol. Conserv. 191:436–443. 10.1016/j.biocon.2015.07.035

[saag025-B108] Speight MCD. 2024. Species accounts of European Syrphidae, 2024. Syrph the Net, the database of European Syrphidae (Diptera). Vol. 115. Syrph the Net publications., p. 381.

[saag025-B109] Srivathsan A , Ang Y, Heraty JM, et al 2023. Convergence of dominance and neglect in flying insect diversity. Nat. Ecol. Evol. 7:1012–1021. 10.1038/s41559-023-02066-0 37202502 PMC10333119

[saag025-B110] Srivathsan A , Hartop E, Puniamoorthy J, et al 2019. Rapid, large-scale species discovery in hyperdiverse taxa using 1D MinION sequencing. BMC Biol. 17:96–20. 10.1186/s12915-019-0706-9 31783752 PMC6884855

[saag025-B111] Srivathsan A , Lee L, Katoh K, et al 2021. ONTbarcoder and MinION barcodes aid biodiversity discovery and identification by everyone, for everyone. BMC Biol. 19:217. 10.1186/s12915-021-01141-x 34587965 PMC8479912

[saag025-B112] Srivathsan A , Sha JCM, Vogler AP, et al 2015. Comparing the effectiveness of metagenomics and metabarcoding for diet analysis of a leaf-feeding monkey (*Pygathrix nemaeus*). Mol. Ecol. Resour. 15:250–261. 10.1111/1755-0998.12302 25042073

[saag025-B113] Stephen WP , Rao S. 2007. Sampling native bees in proximity to a highly competitive food resource (Hymenoptera: Apiformes). J. Kans. Entomol. Soc. 80:369–376. 10.2317/0022-8567(2007)80[369:SNBIPT]2.0.CO;2

[saag025-B114] Stireman JO III, , Cerretti P, Whitmore D, et al 2012. Composition and stratification of a tachinid (Diptera: Tachinidae) parasitoid community in a European temperate plain forest. Insect Conserv. Diversity 5:346–357. 10.1111/j.1752-4598.2011.00168.x

[saag025-B115] Stork NE. 2018. How many species of insects and other terrestrial arthropods are there on Earth? Annu. Rev. Entomol. 63:31–45. 10.1146/annurev-ento-020117-043348 28938083

[saag025-B116] Sutherland WJ , Roy DB, Amano T. 2015. An agenda for the future of biological recording for ecological monitoring and citizen science. Biol. J. Linn. Soc. Lond. 115:779–784. 10.1111/bij.12576

[saag025-B117] Thompson A , Frenzel M, Schweiger O, et al 2021. Pollinator sampling methods influence community patterns assessments by capturing species with different traits and at different abundances. Ecol. Indic. 132:108284. 10.1016/j.ecolind.2021.108284

[saag025-B118] Truett GE , Heeger P, Mynatt RL, et al 2000. Preparation of PCR-quality mouse genomic DNA with hot sodium hydroxide and tris (HotSHOT). Biotechniques. 29:52, 54. 10.2144/00291bm09 10907076

[saag025-B119] Uhler J , Haase P, Hoffmann L, et al 2022. A comparison of different Malaise trap types. Insect Conserv. Diversity 15:666–672. 10.1111/icad.12604

[saag025-B120] Virgilio M , Jordaens K, Breman FC, et al 2012. Identifying insects with incomplete DNA barcode libraries: African fruit flies (Diptera: Tephritidae) as a test case. PLoS One. 7:e31581. 10.1371/journal.pone.0031581 22359600 PMC3281081

[saag025-B121] Wang WY , Srivathsan A, Foo M, et al 2018. Sorting specimen-rich invertebrate samples with cost-effective NGS barcodes: validating a reverse workflow for specimen processing. Mol. Ecol. Resour. 18:490–501. 10.1111/1755-0998.13567 29314756

[saag025-B122] Wang Z , Zeng J, Meng W, et al 2021. Out of sight, out of mind: public and research interest in insects is negatively correlated with their conservation status. Insect Conserv. Diversity 14:700–708. 10.1111/icad.12499

[saag025-B123] Westerberg L , Berglund H-L, Jonason D, et al 2021. Color pan traps often catch less when there are more flowers around. Ecol. Evol. 11:3830–3840. 10.1002/ece3.7252 33976778 PMC8093746

[saag025-B124] Westphal C , Bommarco R, Carré G, et al 2008. Measuring bee diversity in different European habitats and biogeographical regions. Ecol. Monogr. 78:653–671. 10.1890/07-1292.1

[saag025-B125] Wickham H. 2016. ggplot2: elegant graphics for data analysis. Springer-Verlag. https://ggplot2.tidyverse.org.

[saag025-B126] Wood TJ , Holland JM, Goulson D. 2015. A comparison of techniques for assessing farmland bumblebee populations. Oecologia 177:1093–1102. 10.1007/s00442-015-3255-0 25676106

[saag025-B127] Yeo D , Puniamoorthy J, Ngiam RWJ, et al 2018. Towards holomorphology in entomology: rapid and cost-effective adult–larva matching using NGS barcodes. Syst. Entomol. 43:678–691. 10.1111/syen.12296

[saag025-B128] Yeo D , Srivathsan A, Puniamoorthy J, et al 2021. Mangroves are an overlooked hotspot of insect diversity despite low plant diversity. BMC Biol. 19:202. 10.1186/s12915-021-01088-z 34521395 PMC8442405

[saag025-B129] Yi Z , Jinchao F, Dayuan X, et al 2012. A comparison of terrestrial arthropod sampling methods. J. Resour. Ecol. 3:174–182. 10.5814/j.issn.1674-764x.2012.02.010

