# Supplementary material for: Beyond conventional pollinators: advancing diversity assessments of neglected insect taxa
Source: Ann Entomol Soc Am. 2026 Aug 5;119(5):372–85. doi: 10.1093/aesa/saag025 (PMC13562752; doi:10.1093/aesa/saag025)
Supplement: Supplementary file 1 [file saag025_supplementary_data.zip › Figure_S2.pdf]

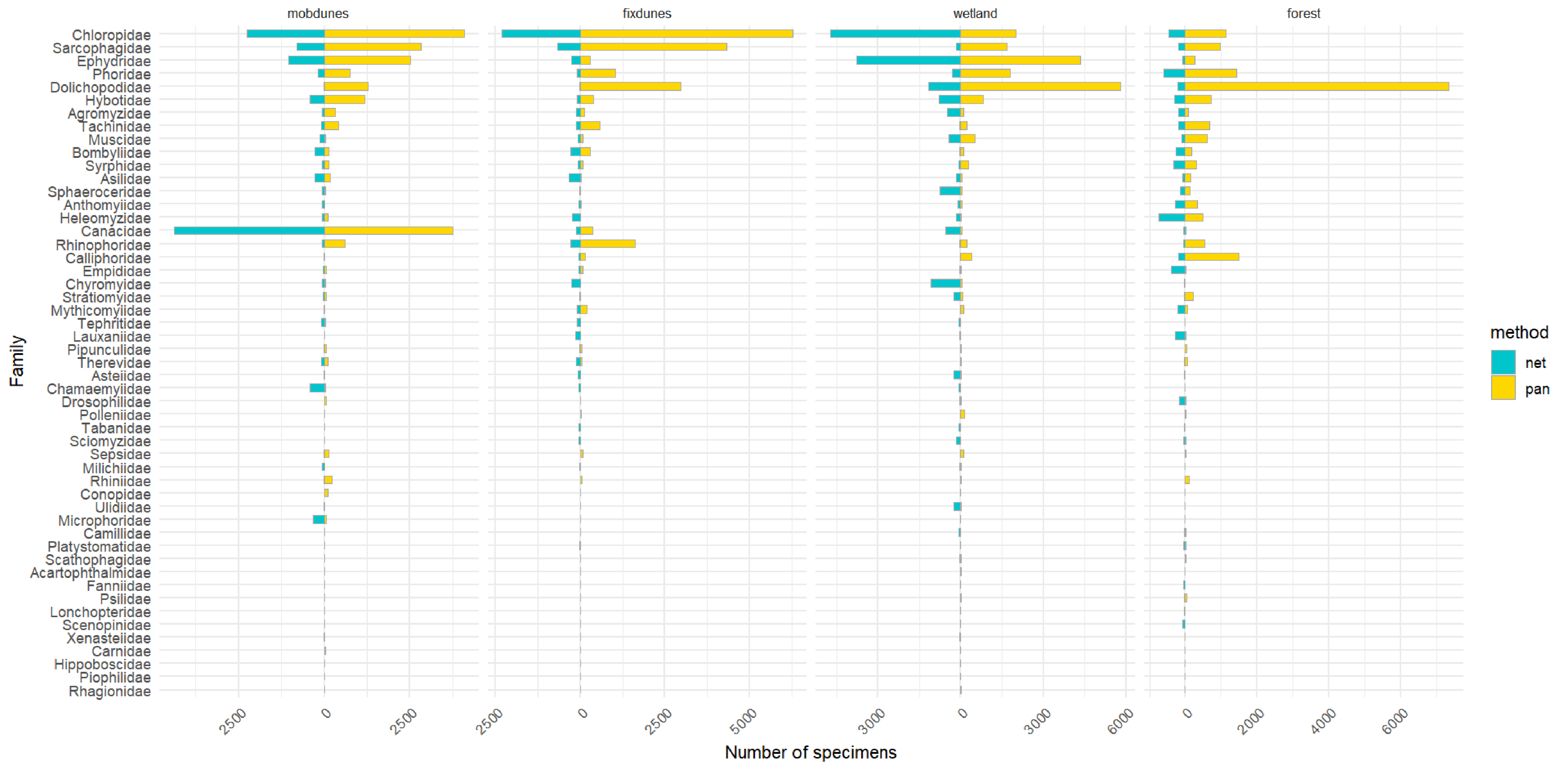
mobdunes
fixdunes
wetland
forest
Family
Chloropidae
Sarcophagidae
Ephydridae
Phoridae
Dolichopodidae
Hybotidae
Agromyzidae
Tachinidae
Muscidae
Bombyliidae
Syrphidae
Asilidae
Sphaeroceridae
Anthomyiidae
Heleomyzidae
Canacidae
Rhinophoridae
Calliphoridae
Empididae
Chyromyidae
Stratiomyidae
Mythicomyiidae
Tephritidae
Lauxaniidae
Pipunculidae
Therevidae
Asteiidae
Chamaemyiidae
Drosophilidae
Polleniidae
Tabanidae
Sciomyzidae
Sepsidae
Milichiidae
Rhiniidae
Conopidae
Ulidiidae
Microphoridae
Camillidae
Platystomatidae
Scathophagidae
Acartophthalmidae
Fanniidae
Psilidae
Lonchopteridae
Scenopinidae
Xenasteiidae
Carnidae
Hippoboscidae
Piophilidae
Rhagionidae
2500
0
2500
2500
0
2500
5000
3000
0
3000
6000
0
2000
4000
6000
Number of specimens
method
net
pan
